# Dr. Warren on Chloroform

**Published:** 1849-05

**Authors:** 


### DR. WARREN ON CHLOROFORM.

Dr. Warren, whose interesting work on Etherization we noticed some time since, has published a second tract on this subject, in which he gives the results of professional experience with anæsthetics down to the present time. From all that has occurred, he is brought to the conclusion that the ethers ought to be substituted for chloroform, Chloric ether was brought by Dr. W. to the notice of the medical association of Baltimore, last spring, as an agent which seemed to promise well for inducing anæsthesia. He would not use anæsthetics at all for trifling operations, and when deemed admissible would employ the ethers. He further cautions against excluding atmospheric air in the inhalations; recommends bleeding in cases where the narcotism is to be carried very far; to watch the pulse and respiration; not to administer these agents upon a full stomach; and, in operations not very severe, to produce only insensibility to pain without abolishing the intellectual functions. His "confidence in the beneficial effects of ether, in surgical operations, is undiminished;" but he adds, that if he were compelled to substitute chloroform, he would "do so with much anxiety."

Enough will be found in this treatise to awaken anxiety in all cautious practitioners about to administer this powerful agent. Nevertheless, we are still advocates for its cautious use. While it has been fatal in the hands of dentists and surgeons, and occasionally when imprudently inhaled by persons for the pleasurable sensations excited by it, we are yet to hear of the first instance in which death has followed its use by the physician or accoucheur. This, we think, cannot be accounted for by the comparatively limited number of the character of the cases, but depends upon the more gradual manner in which anæsthesia is brought about in such practice.

We have read Dr. Warren's second publication with great pleasure, and commend it to all readers wishing details on the subject.

